# Belongingness challenged: Exploring the impact on older adults during the COVID-19 pandemic

**DOI:** 10.1371/journal.pone.0276561

**Published:** 2022-10-20

**Authors:** Elfriede Derrer-Merk, Scott Ferson, Adam Mannis, Richard P. Bentall, Kate M. Bennett

**Affiliations:** 1 Department of Psychology, University of Liverpool, Liverpool, United Kingdom; 2 Institute for Risk and Uncertainty, School of Engineering, University of Liverpool, Liverpool, United Kingdom; 3 School of Engineering, University of Liverpool, Liverpool, United Kingdom; 4 Department of Psychology, University of Sheffield, Sheffield, United Kingdom; King Abdulaziz University, SAUDI ARABIA

## Abstract

**Objectives:**

The sense of belonging is a fundamental human need. Enacting it through face-to-face social activities was no longer possible during the COVID-19 pandemic. In this study, we investigate how the sense of belonging, and how it is enacted, changed longitudinally amongst older adults in the UK. In addition, we examine the interplay of the sense of belonging and resilience over time.

**Methods:**

We employed a longitudinal qualitative research design to explore the experiences of older adults during one year of the COVID-19 pandemic (April 2020-April 2021). The analysis was undertaken with constructivist grounded theory.

**Findings:**

Before the pandemic older adults were free to engage in social relationships with family and friends, often enacted within social activity groups where they felt valued and gained positive experiences. During the pandemic face to face enactment of belongingness was reduced; adjustments needed to be made to maintain the sense of belonging. The experience of older adults was heterogeneous. We examine three themes. First, how belongingness was enacted prior to the pandemic. Examples include: family holidays, visiting each other, sports activities, eating with friends and family, and visiting cultural events. Second, how participants adapted and maintained their social involvement. Examples include: distanced face-to-face activities; and learning new technology. Third, for some, a belongingness gap emerged and persisted. There was an irretrievable loss of family members or friends, the closure of social groups, or withdrawal from groups as priorities changed. As a consequence, of challenged belongingness, participants expressed increased loneliness, anxiety, social isolation, frustration and, feelings of depression. For many, the disrupted sense of belonging no longer fostered resilience, and some previously resilient participants were no longer resilient.

## Introduction

By January 2022, the severe acute respiratory syndrome coronavirus 2 (SARS-CoV-2), known as COVID-19, has persisted for two years, changing the lives of people worldwide. Health and safety measures have been introduced to stop the virus from spreading. In the UK, for example, lockdowns, shielding advice, social distancing (staying 2m apart), wearing face masks, and using hand sanitizer regularly were imposed at various times since March 2020 [1]. Despite the international effort to stop or slow down the spread of the virus, including a new COVID-19 vaccine roll out in January 2021, the death toll has risen dramatically. In the UK, cumulative mortalities, judged by COVID-19 recorded on death certificates, had reached about 120 000 people by January 2021 [2]. In particular, older adults were at higher risk from the COVD-19 pandemic, with greater disease severity and higher mortality compared to younger people [3]. Older adults often experienced comorbidities and potentially a declined immune system [4]. Several authors have stressed that this combination, leading to a greater physical vulnerability, created a psychosocial threat from SARS-CoV-2 [5–7]. For some people, the COVID-19 health and safety measures led to increased mental health problems, such as increased anxiety, feelings of depression, social isolation, and loneliness [7–18].

**Fig 1:**
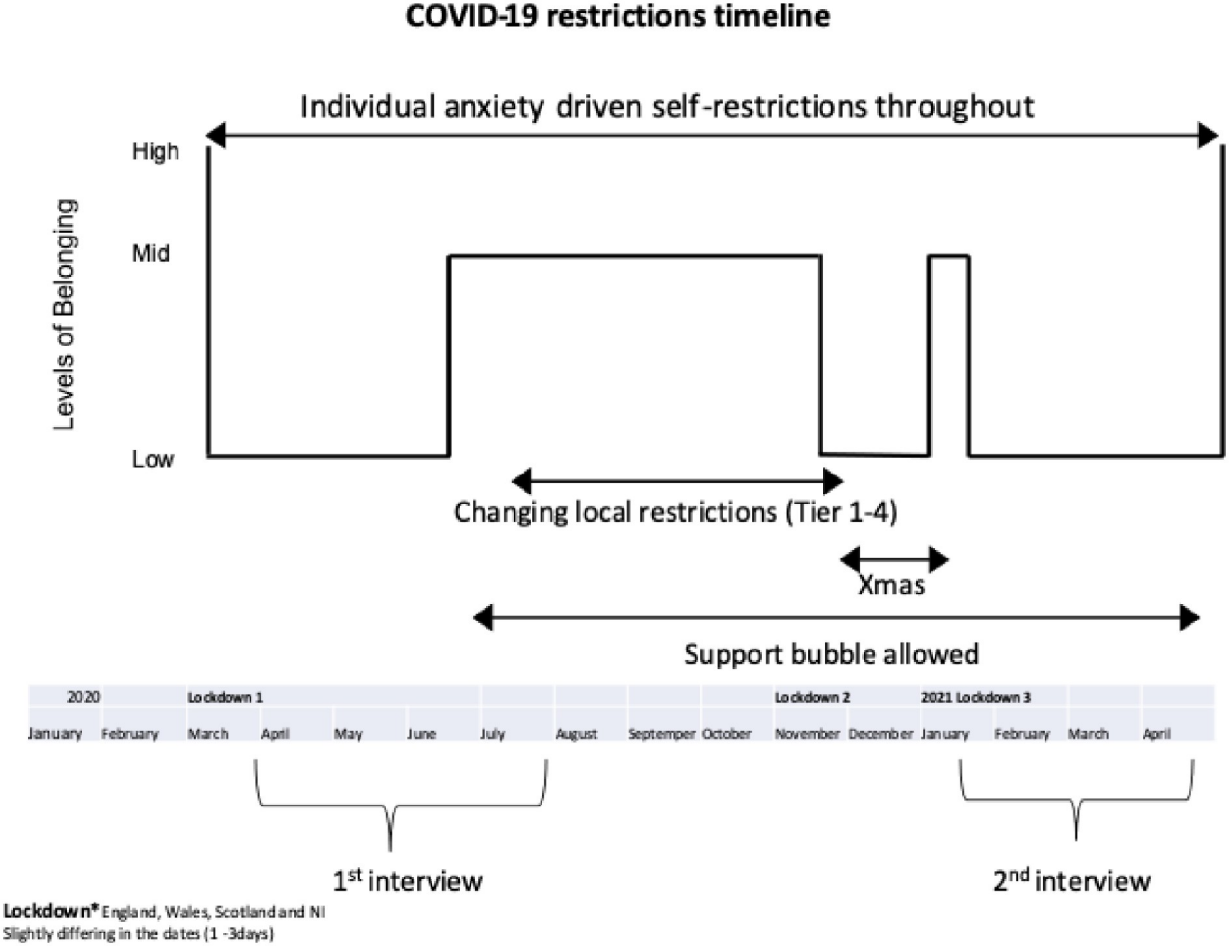
Enacted sense of belonging: Timeline (April 2020-April 2021).

The COVID-19 pandemic had additional impacts, one of which had been the disruption of the sense of belonging. Relationships with those who are proximate, physically close with whom people have face-to-face contact, have been disrupted most which, in turn, has challenged the sense of belonging for older adults. Our interest in the sense of belonging, was woken by participant M15W1 who told us: ‘but we’ve never been totally removed from the family’ (M15) (for fuller discussion see [1]). Thus, we were interested in how the need to belong appeared, spontaneously, in the data and how it changed over time. A timeline of the lockdowns in the UK and their implications for enacting the sense of belonging can be seen in Fig 1.

In the theory of the Hierarchy of Human Needs, Maslow [19] argued that the need to love and to belong to social groups was an essential human need. Baumeister and Leary [20] subsequently reviewed a wide range of evidence to demonstrate that “human beings have a pervasive drive to form and maintain at least a minimum quantity of lasting, positive, and significant interpersonal relationships” (p. 497). The main providers of belongingness are relationships with family members and friends. These relationships can be maintained by: a) frequent interactions and b) ‘actively pleasant’ interactions, including being concerned for ‘each other’s welfare’ in a stable framework [20] p.1). In a meta-analysis, Hesse et al. [21] found that the human need to have satisfactory bonds and relationships to other humans was essential for people’s well-being and highlighted the quality of the relationships as fundamental. Mutual exchange of affection enables humans to develop and maintain social bonds and in consequence their sense of belonging. Positive physical contact such as hugging and cuddling is also an important component of belongingness both psychologically and physiologically [22–24]. Additionally, Lim et al. [25] found that individuals need to belong is important for wellbeing. They found that the need to belong and loneliness are two independent coexisting concepts of a dual continuum encompassing the aspects of socially fulfilled, socially searching, socially indifferent and socially distressed individuals [25]. Despite the complexity and how these concepts are interwoven, we are focusing in this study on the sense of belonging based on the spontaneously reported experiences of older adults (more details see [53]). Thus, deprivation of social and physical contact may reduce the sense of belonging, and in turn lead to increased social isolation, loneliness, and reduced wellbeing. Resent research highlighted the risk of suicide when the need of belonging is not met [26, 27].

One aspect of psychological well-being which is particularly relevant during a crisis such as the COVID-19 pandemic is resilience. Despite increased research effort with quantitative and qualitative approaches, psychological resilience is still a contested term. Only two common components (adversity and positive adaptation) are common ground were researchers can agree on [28, 29]. There is currently no consensus in defining the concept of resilience nor how to operationalise it [28–33]. Cosco et al. [28] identified three methodological ways for the operationalisation: psychometric driven, definition driven and data driven. Qualitative measurement methods were also found in a World Health Organisation review by South et al., [34]. In 2009, a UK network considered resilience across the lifespan (http://resilience.bangor.ac.uk/index.php.en). The network produced a concept analysis [35] and from this concept analysis produced the following definition:

Resilience is the process of negotiating, managing, and adapting to significant sources of stress or trauma. Assets and resources within the individual, their life, and environment facilitate this capacity for adaptation and ‘bouncing back’ in the face of adversity. Across the life course, the experience of resilience will vary [35]

(p.220).

In this study we use this definition, later operationalised by Donnellan et al. [36], which is described in the methods. Also developed from the work of the network is the Ecological Model of Resilience [35] which has been successful evaluated across a number of domains, and methods [35–37]. Thus, this study builds on the work of the network in the context of COVID-19 using a data-driven qualitative approach to operationalise resilience (in detail see below) [36]. This enables us to understand the experiences of older adults during the pandemic and fosters recommendations, based on the lived experiences, to improve policies and practise by enhancing health and well-being.

**Fig 2:**
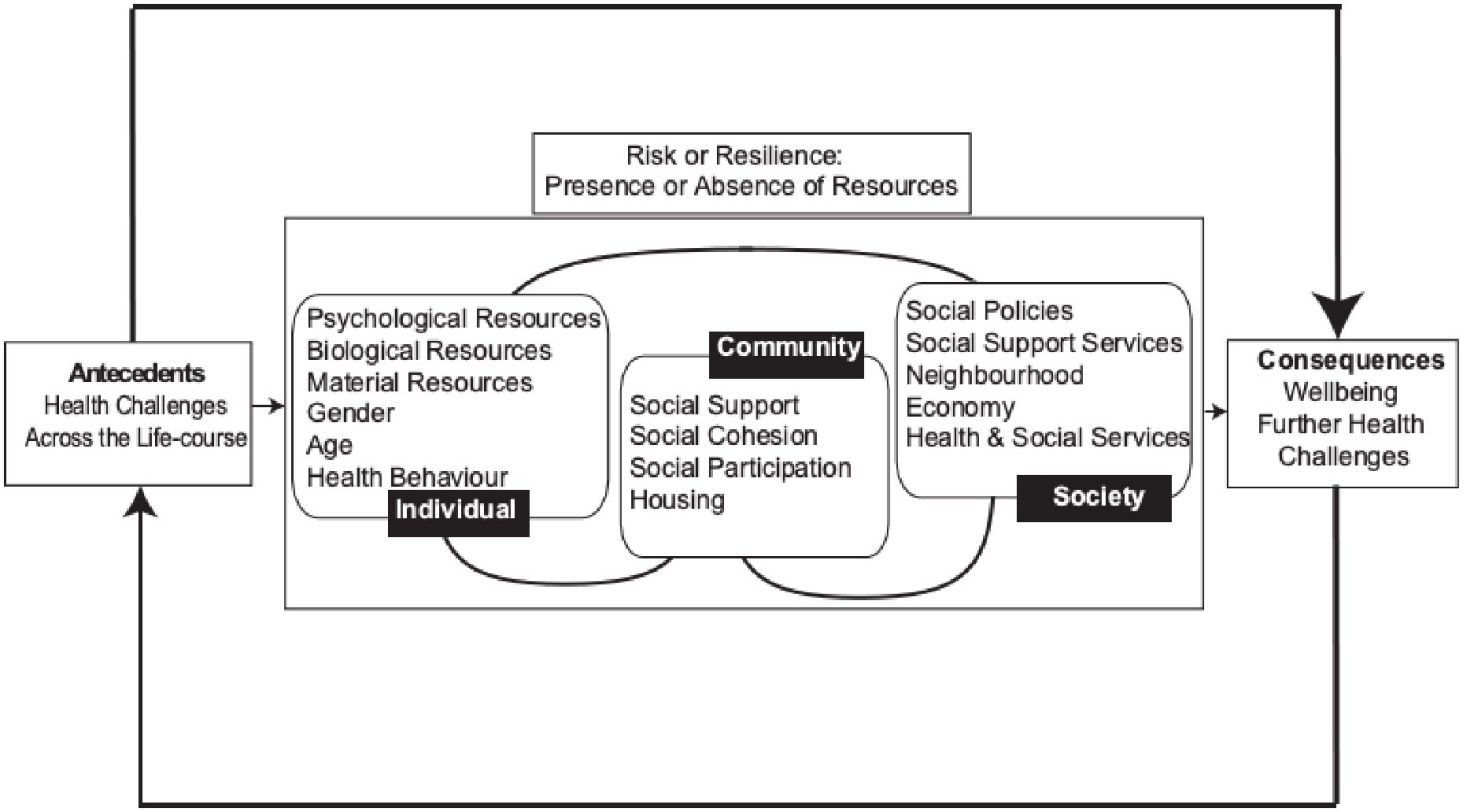
Ecological model of resilience [35].

The Ecological Model of Resilience (Fig 2) proposes that individual, community, and societal resources were important in facilitating, and indeed hindering resilience [35–37]. This model has also been successfully tested before the pandemic [38] and recently during the COVID-19 pandemic in an Italian Study [39]. This Ecological Model of Resilience includes individual resources (e.g. personality, life philosophy), community (e.g. social support, family, community cohesion) and societal (e.g. health, welfare services, social policy, religion). The sense of belonging is a cross-cutting component of individual, community, societal resources which can foster or hinder resilience. (see also [40]). Thus, we are interested in exploring these relationships further, in particular how they have changed during the COVID-19 pandemic when these resources where limited.

The pandemic provides an opportunity not only to examine the experience of belongingness but also to test whether changes in belongingness impact resilience. Hence, in this paper we use qualitative data collected during the pandemic to answer two questions:

1. How did older adults in the UK experience belongingness during the pandemic?
2. How does the sense of belonging impact resilience over the course of the first year of the pandemic?

To our knowledge, no one has previously reported a longitudinal qualitative investigation of this kind in the UK.

## Design and methodology

This study uses a longitudinal qualitative research methodology (LQR). In a systematic review, Nevedal et al. found that the longitudinal studies enrich and deepen gerontological research and they recommended the use of LQR clarification for this purpose [41]. Longitudinal qualitative research is a particularly useful approach for investigating social phenomena, such as the COVID-19 pandemic [42]. It provides a unique opportunity to understand the lived experiences of older adults during the COVID-19 pandemic (April 2020- April 2021). There is no gold standard for LQR methodology but it is important to constantly assess the data and maintain flexibility [43–45]. These strategies are reflected in the constructivist grounded theory by Charmaz [46–48] which we employ. Our underlying LQR analysis followed the ontological and epistemological position that there are multiple truths and realities about phenomena and that society and humans construct their reality. Based on this ontological and epistemological position our intention is to develop theory, rather than to test existing theories. Thus, our findings are grounded in the language of our participants [48–51]. Charmaz’s [47] recommends that the analyses must be co-constructed between researcher and participants and aims to develop a theory grounded in the data [46]. The analysis is inductive and iterative, using constant comparison. Details of the analytical method are detailed below.

### Sample

The COVID-19 Psychological Research Consortium Study (C19PRC), formed in March 2020, aimed to investigate the psychological, social, and economic impacts of the COVID-19 pandemic (https://www.sheffield.ac.uk/psychology-consortium-covid19). This longitudinal, internet panel survey assessed: (1) COVID-related knowledge, attitudes, and behaviours; (2) the occurrence of common mental health disorders; as well as the role of (3) psychological factors; and (4) social and political attitudes in influencing the public’s response to the pandemic (more details see [52]). The data that support the findings of this study have underlying ethical data protection and are not openly accessible but are available on request. More details can be found in the open science framework qualitative studies [53]. Quota sampling was used to recruit a nationally representative (age, sex, and household income) sample of adults (N = 2025). The consortium was not confined to the UK but included collaborations from Ireland, Italy, and Spain. As one part of this program, we aimed to understand the lived experience of adults during the pandemic in a qualitative study (see below).

### Recruitment

Participants in the UK were recruited from participants in the main C19PR study (for more details see [1, 53]), who were asked if they would be willing to be approached to participate in add-on studies. Those who consented were grouped into three subsamples and contacted by email to ask if they would be willing to be interviewed: older adults (≥, 65 years old); adults (18–64); and pregnant women and parents with children under the age of 1 year old. This paper focuses on the sample of older adults. In the larger study, there were 287 participants aged 65 and over and we contacted 86 of those, and 33 participants agreed to participate (38.4% participation rate) in the first wave of interviews (Wave 1) (Table 1). Recruitment was halted when theme saturation was reached (for more details see [47, 48, 54]). The interview team approached potential participants in batches of 5–10 and ceased recruitment once theme saturation had been reached. Wave 1 interviews were conducted between April and July 2020 and participants were compensated with a £10.00 voucher from The University of Liverpool. Second, follow-up interviews (Wave 2) took place between January and April 2021. All previous participants were invited to take part and 29 out of 33 (88% participation rate) participated, with only four male participants declining to take part in the follow-up interview (Table 1). Again, the participants were compensated with a £ 10.00 voucher from the University of Sheffield.

**Table 1:** Participants demographics Wave 1 and Wave 2 (including classification of resilience).

	UK Wave 1 (n = 33)			Resilient	UK Wave 2 (n = 29)		
	ID	Age*	Marital Status*		ID	Resilient	Change
**Men living Alone**	**M1**	**67**	**Widowed** **	**NR**	**M1**	**NR**	
	**M3**	**74**	**Never married**	**R**	**M3**	**R**	
	**M7**	**83**	**Divorced**	**R**			
	**M8**	**73**	**Divorced**	**NR**	**M8**	**Nr**	
	**M10**	**75**	**Divorced**	**R**	**M10**	**R**	
	**M14**	**77**	**Widowed**	**R**	**M14**	**NR**	**R ->NR**
**Men not living Alone**	**M2**	**73**	**Married**	**NR**			
	**M4**	**71**	**Married**	**R**	**M4**	**NR**	**R ->NR**
	**M5**	**75**	**Married**	**R**			
	**M6**	**71**	**Married**	**R**	**M6**	**NR**	**R ->NR**
	**M9**	**64**	**Married**	**R**	**M9**	**R**	
	**M11**	**70**	**Married**	**R**			
	**M12**	**66**	**Married**	**R**	**M12**	**R**	
	**M13**	**77**	**Civil partnership**	**NR**	**M13**	**NR**	
	**M15**	**72**	**Married**	**R**	**M15**	**R**	
**Women living Alone**	**F2**	**78**	**Never married**	**R**	**F2**	**R**	
	**F4**	**75**	**Widowed**	**R**	**F4**	**R**	
	**F6**	**67**	**Widowed**	**R**	**F6**	**R**	
	**F8**	**66**	**Separated**	**R**	**F8**	**NR**	**R ->NR**
	**F9**	**82**	**Widowed**	**R**	**F9**	**NR**	**R ->NR**
	**F10**	**65**	**Never married**	**R**	**F10**	**R**	
	**F11**	**73**	**Divorced**	**NR**	**F11**	**NR**	
	**F12**	**68**	**Divorced**	**R**	**F12**	**NR**	**R ->NR**
	**F14**	**76**	**Separated but still married**	**R**	**F14**	**NR**	**R ->NR**
	**F15**	**73**	**Divorced**	**NR**	**F15**	**NR**	
	**F16**	**73**	**Divorced**	**R**	**F16**	**R**	
	**F17**	**70**	**Widowed**	**R**	**F17**	**R**	
**Women not living Alone**	**F1**	**65**	**Married**	**R**	**F1**	**R**	
	**F3**	**71**	**Married**	**R**	**F3**	**R**	
	**F5**	**65**	**Divorced**	**R**	**F5**	**R**	
	**F7**	**65**	**Married**	**R**	**F7**	**NR**	**R ->NR**
	**F13**	**68**	**Married**	**NR**	**F13**	**R**	**NR->R**
	**F18**	**66**	**Married**	**R**	**F18**	**R**	

R = resilient, NR = not resilient,

* age and marital status W1

** W2 living with daughter

### Sub-sample older adults

The study aimed to interview participants at two different time points during the COVID-19 pandemic (Fig 1). This paper utilises the sample age 65+ (range 65–83; mean age = 71, SD = 5) in Wave 1, as described above. We recruited based on the living situation, rather than marital status because that was more potentially important in the context of lockdowns. In Wave 1 we recruited 6 men living alone, 9 men not living alone, 12 women living alone, and 6 women not living alone. In Wave 2, 4 men were living alone, 7 men not living alone, 12 women living alone, and 6 women not living alone (age range 65–83, mean = 72, SD = 5). For more details see Table 1. Note that details on the classification process are described later.

### Ethical approval

The ethical approval for the national representative study was granted by the University of Sheffield University (ref: 033759). The qualitative sub-study was approved by the University of Liverpool (ref: 7632–7628).

### Data collection

The data collection was undertaken using a semi-structured interview schedule, at two-time points, via in-depth interviews (Fig 1). Interviews were undertaken remotely via telephone, or by remote conferencing platforms, and lasted between 30 and 90 min. The interviews were audio-recorded and externally transcribed verbatim. Before beginning the interview, respondents were sent an information sheet via email and a consent form to read and sign. Confidentiality and anonymity were assured. The interviews were not tightly structured; rather the aim was to learn what was important to the participants. The approach takes “participants’ views and voices as integral to the analysis—and its presentation” (sic) [46] (p.402). Two broad questions were asked, ‘How did you feel?’ and ‘What did you do?’ The interviews led participants chronologically through their experiences of the COVID-19 pandemic at Wave 1. They were asked about their lives before the pandemic, then about their lives during the pandemic, and finally about their thoughts about their future after the pandemic. They were also asked about their experiences as older adults, views about their communities, local and central government, and social and traditional media (see more details [53]). In Wave 2 we also asked what had changed since the last interview, what differences they found between the COVID-19 waves, and changes in the way they communicated with family and friends (more details see [53]).

### Analysis

We applied in this study a three-stage hybrid method.

1. First, we analysed and coded each interview from W1 and W2 independently concerning the sense of belonging as described below. We began by reading and coding the interviews line-by-line, summarised in a codebook. This process was reflective: as new topics emerged, they were looked for in earlier parts of the interview. Followed by focused coding, the first author wrote memos helping to construct theoretical categories and ensuring reflexivity and analytical thinking [46]. In the final stage, we then co-constructed overarching themes from both interview times, which reflected the changes in the sense of belonging and resilience over time.
2. Second, we identified and classified participants as resilient or not resilient independently for both interview waves (Table 1). We used Donnellan et al.’s (2015) [36] operationalisation of Windle’s definition to classify participants: “A) participants experienced significant challenge: COVID-19 pandemic. B) participant shows no sign of (di)stress and no medication to maintain emotional wellbeing. C) participants maintained meaningful life and satisfaction (a sign of bouncing back). D) participant is actively participating in life (a sign of managing) (e.g. video call instead of face to face). E) the current life is seen as positive (a sign of adaptation)” [36] (p.4). The classification was undertaken by two members of the team independently and any discrepancies in classification were resolved through discussion with the wider team. The significant challenge applies to all participants as this study was undertaken during the COVID-19 pandemic. This classification enables us to identify participants who may be more vulnerable at this time.
3. Third, we examined in what ways, if any, the sense of belongingness impacted resilience over the first year of the pandemic.

## Findings

The changes in proximity to loved ones and friends impacted the participant’s sense of belonging and resilience. The sense of belonging, known as a major resource and protective factor for resilience, had become fragile during the time of COVID-19. However, 26 out of 33 participants were resilient in the first wave of interviews. After one year 21 out of 29 remained resilience. Five participants remained non-resilient between waves. W1 and W2, three of whom experienced a disrupted sense of belonging which impacted their resilience. Only one participant became resilient, but this could not be linked to the sense of belonging.

The hybrid analyses highlighted the following themes which related belongingness to resilience: enacted belongingness pre-pandemic; challenged belongingness; and gaps of belongingness.

### Enacted belongingness pre-pandemic

Prior to the pandemic participants experienced a sense of belonging by enjoying other people’s company, often in proximity, exchanging support, and expressing concern for each other’s welfare. 26 out of 33 participants who appeared resilient (Table 1) in Wave 1 maintained a life of meaning, participated actively in social activities and had a positive attitude to life. We will discuss in turn the enacted sense of belonging and its impact on resilience from family, friends, and group activities. Participants identified as follows: F (woman); M (man); W (Wave); R (resilient); NR (non-resilient).

#### Family

The sense of belonging to the family was often expressed when participants talked about the joy of grandparenting as F12 discussed (W1 R, W2 NR):

I’m involved in my grandchild’s child care. (…). With my daughter it mainly involves the grandchild, so we would have days out to… I love taking my granddaughter out to museums and art galleries in other towns and cities, so we do a lot of like days out

(F12 W1)

Similarly, a list of activities F15 provides evidence of how the sense of belonging was maintained. However, despite her involvement in family activities, she reported elsewhere the use of medication to maintain her mood, and therefore we classified here as non-resilient (W1, W2 NR):

With my family, we tend to go out for days out to local attractions—the seaside, the beach, wildlife parks, amusement parks.

(F15 W1)

#### Friends and group activities

Many participants spoke of the importance of friends and activities in groups. These activities and shared common interests provided pleasure and a sense of belonging. Many participants participated in more than one group activity as M4 discussed. He spent almost every day with friends and intertwined group activities. His belongingness was enriched: by an intensive social life and is fundamental to his resilience (W1 R, W2 NR):

We have such a social life, we’re very rarely in the evening. In the four weeks before lockdown, we happened to have a show so we were doing a show at the theatre for seven nights, but the 28 evenings before lockdown, we were only at home for five of those evenings. (…). My wife is into singing and musical theatre and I join in with that with her from time to time. I’m into golf, I play golf twice a week.

(M4 W1)

Another participant F8, recognised, during the first lockdown, how important her involvement in group activities had been pre-pandemic. This active participation contributes to her resilience (W1 R, W2 NR):

I have a fair few activities outside the home which involves meeting in groups, things like singing in a choir, going walking with friends, doing Pilates, volunteering in a charity shop (…) so it’s made me realise how important the things I do are.

(F8 W1)

The sense of belonging was enacted through meeting family and friends regularly and enjoying being part of groups or spending time together. Many participants spoke of their happy life pre-pandemic. As people met in person, technology to bridge the gap of social disconnectedness was not often mentioned, although the telephone was used when friends and family members outside the country were contacted. This sense of belonging and the available resources such as family and group activities contributed to resilience before the pandemic.

### Challenged belongingness (April 2020- April 2021)

At the beginning of the COVID-19 pandemic, the face-to-face methods used to enhance and maintain belongingness with family members and friends came to a halt. As time went on, the 1st lockdown eased, but social distancing measures (staying 2m apart, wearing masks, etc.) still restricted proximate activities. As the pandemic waxed and waned over the following year, accompanied by changes in social distancing rules, participants experienced challenges to their sense of belonging (Fig 1). With the disruption of the sense of belonging many participants were unable to maintain their resilience (Table 1).

However, despite the uncertainty and the threat of the virus, some participants found ways of adapting, although they often noted that these new ways were not good substitutes for face-to-face contact. We discuss in turn the changes of belonging and resilience related to family, friends and group activities, and technology.

#### Family

The ups and downs of meeting and staying apart from family members is described by F5, who noted, first, how difficult her experience had been and, second, how the vaccine has given her hope: She demonstrated resilience in W1 and W2, her creativity to adapt to the restrictions e.g. sitting on the pavement and the W2 actively participating in life (childcare) are clear signs of resilience. The quote in turn is evidence of her mental wellbeing (W1, W2 R):

I’ve been in and out of my daughter’s bubble because I live on my own and I wasn’t going in her house since Christmas lockdown, January lockdown, but now that I’ve had the vaccine we’ve agreed that I can… I’m in their child care bubble and we’ve agreed I can go back into the house, so that feels like a bit of a step towards normal, because before I was just sitting outside on the pavement.

(F5 W2)

Christmas is a special season of the year when family members usually meet and share the joy of the festive days. However, Christmas 2020 was different in the UK: only three households were allowed meet for one day as M12 discussed: He had no sign of distress and maintained meaning and satisfaction in his life in both interviews (W1, W2 R):

We had Christmas day with my son, daughter-in-law, and my daughter-in-law’s mother and brother who both live in [city], so that was six people, three households, and that was it. We’ve seen them since, but not indoors obviously.

(M12, W2)

Despite the option to meet family members indoors on Christmas day (25^th^ of December), one participant F9 could not see her family as they live far away. She expressed her frustration and the impact on her well-being. Not only that her wellbeing was impacted by the missing proximity of loved ones, but it also impacted her resilience and this changed from W1 resilient to W2 not resilient as she was elsewhere reporting using medication (W1 R, W2 NR):

I’m lonely. I’m not seeing family. I’m not seeing friends. I haven’t been anywhere since about the end of October. I had Christmas on my own, my birthday on my own. Everyone else’s birthdays, I couldn’t go to celebrate. It’s been hard. (…) I think being shut in all the time and just being on your own, it does make you, not nervous but apprehensive.

(F9 W2)

The lack of proximity hit many participants hard like F7 expressed. The disrupted sense of belonging and missing resources for resilience made it difficult to manage a stressful situation. She disclosed her signs of distress and was identified as not resilient in W2 (W1 R, W2 NR):

Obviously missing my daughter. Well, lonely I guess, from that point of view, or sad from the point of view of missing her. Anxious, I don’t know; yes, anxious that the Government’s not going to do as much as it could do.

(F7 W2)

Another participant F12 talked about her experience with limited time to live and missing the proximity of her family in particular the grandchildren. She had been identified as resilient in W1 but due to the missing protective factors of seeing the family face to face, it changed to not resilient in W2 (W1 R, W2 NR):

But I have found it very difficult, because I think as you get older you know you’ve only got, I don’t know, 10/15 good years of life left and to take a year out, that’s a high percentage of that time, high proportion of that time and I’ve got family overall that I can’t see and I’m missing my grandchildren that I can’t see.

(F12 W2)

Another participant F8 received traumatic news and expressed her pain at the approaching loss of her beloved brother. In W1 she was able to maintain an active life and took part in online courses, but her brother’s diagnosis took her protective resource away which affected her wellbeing. (W1 R, W2 NR):

I have four brothers, one of them, the youngest has just been diagnosed with an advanced stage four brain tumour which is inoperable‥ (…). Well yes, losing family members, because although as a family we’ve never lived in each other’s pocket, we don’t communicate constantly, but there is a very strong bond and the thought of, for example, losing my brother who is of my four brothers my favourite brother, I couldn’t really conceive of it. I still haven’t got my head round it because him not being in the world will completely change things for me.

(F8 W2)

One participant W2 M4 had been involved in activities with family and friends almost every day pre-pandemic and was identified as resilient in W1 but the sudden challenge of belongingness and missing proximity made him suffer (W1 R, W2 NR):

For some people, that may not be a problem but to us, we have missed the social interaction with friends and family.

(M4 W2)

#### Friends and group activities

For many, meeting friends continued to be very important as they were part of the daily social life pre-pandemic. Notably, many participants reported that they had more contact with friends during the pandemic than before. They maintained social activities and lived a meaningful life in both interviews and maintained resilience (W1, W2 R):

I am probably in contact with more people than I would normally be. I would normally meet people. I am possibly in touch with people further away than I would normally be.

(M3 W2)

Similarly, F6 was not impacted by the restrictions since she felt happy on their own. (W1, W2 R):

I am fine. I don’t get depressed. I am quite happy to be on my own. I like to go out and see people, but I am quite happy on my own.

(F6 W2)

When all clubs closed, the participants’ involvement in group activity was greatly disrupted. M9 expressed his frustration. Despite the frustration he showed no sign of distress and maintained resilience as his family maintained his resource of joy (W1, W2 R):

We gave up golf when it shut down in November. We gave up even meeting in groups of two. The golf course is closed so my secretarial role is null and void at the moment. (…) Everything is on hold. The social activity I do with my friends is play golf, which is curtailed currently.

(M9 W2)

Then he explains what makes him happy:

Everyday occurrences make me happy. My son has a silly sense of humour, (…) He keeps me amused. My wife (…) keeps me amused as well.

(M9 W2)

The motivation to exercise with a group face to face was also very important for F15. She discussed the lack of motivation to exercise on her own. Her sense of belongingness through the gym was no longer accessible during the lockdown. This in turn impacted her resilience. Elsewhere in her interview, she reported using medication to help her cope (W1+W2 NR):

My hobby really was the gym and that’s not happening at all. They have started to do classes online and I’ve tried to do that, but it’s not very motivating. (…) I think I need the company of other people to motivate me to do it.

(F15 W2)

#### Technology

Many participants wanted to be close to family and friends and used the new virtual meeting options to stay connected. However, their experiences were varied. One participant F1 explained how she could do grandparenting remotely this sense of belongingness from the family helped her to maintain resilience (W1, W2 R):

I have enjoyed having a daily Zoom conference with my four-year-old grandson. (…). I feel I am doing childcare remotely!

(F1 W1)

For others, the technical substitute was not easily accessible and F11 struggled to adapt to virtual meetings but used at least text messages to maintain the bonds. She disclosed in the first interview her nervousness about going out and in the second interview reports elsewhere she is drinking more than she used to. These are signs of not being resilient (W1, W2 NR):

I’ve tried a couple of Skype or Zoom calls with the family and, to be honest, I haven’t been able to connect because I’m hopeless. (…) I do use my mobile more, I think. I think I do communicate quite a lot on WhatsApp now, with more of the family than I did before. Yes, most probably that’s all, but not really any improvements on my side.

(F11W2)

The sense of belonging was shaken over the space of one year. Thus, participants needed to find new strategies to enact belongingness. Our findings demonstrate that many participants created new ways of symbolic proximity to maintain belongingness, whilst others struggled. But overall, the change over time of belongingness stretched older adults’ ability to endure the missing interpersonal relationship. Therefore, the loss of resources such as family proximity and group activities, made it hard to maintain resilience over time.

### Gaps of belongingness

The sense of belonging had not only been challenged for many participants during the COVID-19 pandemic but was not replaceable for others. This occurred when participants continued to avoid seeing family and friends even when it was allowed, when loved ones had died or had withdrawn from society, or when participants withdrew themselves from group activities. Some were anxious about getting infected and followed the recommendations of social distancing more strictly than the governments’ advice.

One gap of belongingness occurred when participants could no longer attend activity clubs as they had closed and did not open again. However, we acknowledge the heterogenous experience and impact on resilience.

Despite the closure of the clubs F18 attended in person pre-pandemic, she managed to join the meetings during the pandemic provided online and demonstrated, therefore, signs of active participation. In W2 she reassessed her social priorities, demonstrating maintaining meaningful life and satisfaction and can be identified as resilient (W1, W2 R):

I shall not be going back to everything because I’ve realised that some things are not as important as I thought they were. (…). I don’t want to be involved with that anymore, I don’t want to be on their committee, which I did nothing but moan about and I thought why put yourself through it then.

(F18 W2)

Many participants experienced the death of friends or family members and suffered pain from the loss, as F17 comments. Despite her bereavement, she expressed no current distress and, therefore, we argue she was resilient at both waves (W1, W2 R):

A few months ago, one of my close relatives died due to Covid in India,—and I feel quite miserable about her. She was younger than me, we were very close.

(F17 W2)

Similarly, F 16 was resilient in both interviews, although she had experienced losses in the interim. A sign of managing has been identified when she told she speaks to her sister (W1, W2 R):

I speak to my sister, mmm, every other day probably. (…) I was very upset, particularly when my friend and my brother-in-law died and that was a difficult period.

(F16 W2)

In contrast, another participant F14 spoke about her the multiple losses and the impact they had on her life (W1 R, W2 NR):

Sad because my daughter lives in [place]. I have lost a friend and two relations (…). I haven’t been able to go to the funerals. (…). You could have 30 there (…). I went to one in November and three this year. They are nothing to do with Covid, they are just people who were all older than me so it was inevitable. It didn’t feel… this word I don’t like. Closure. I didn’t think there was any closure because I wasn’t actually there. [upset] Sorry.

(F14 W2)

The sense of belonging was disrupted when activities could not go ahead like for F9’s dancing class. She decided not to continue her beloved hobby as she was hindered by her anxiety-driven thoughts. Thus, she was not resilient (W1 R, W2 NR):

I started going to dancing one afternoon, a tea dance, one afternoon a week and I found that was great, it was giving me exercise, I was meeting people, and of course all that stopped because of COVID and I don’t know if I will ever have… I don’t know what the word is, not strength but I just don’t think I’ll be able to do it again because I won’t be sure that the person dancing with me is not breathing germs on me.

(F9 W2)

M14, who could have joined the family Christmas party but decided not to because he felt safer of staying on his own. Missing the proximity of his loved ones left a gap of belongingness. This in turn led to feelings of depression, and loneliness and the disruption of proximity changed his resilience. (W1 R, W2 NR):

So, I must admit, I have been feeling a bit depressed, at having to be stuck in, with nothing but books and the TV as company. (…). I could have gone to one of my granddaughter’s on Christmas day but I decided it was going to be… Well, since I hadn’t had a first vaccine at least it was going to be doubtful whether I could steer clear of COVID-19 by doing so. So I didn’t go. My other half, my daughter-in-law’s mother, went. She’s now dead. (…). Yes, I suppose I tend to be a bit lonely, being stuck in all the time. Yes, I can pick up the phone and talk to friends, and check up on some of my more elderly friends who aren’t doing too well at the moment, unfortunately, but as the time goes on that’s how it is. (…) No, I’m afraid not much makes me happy at the moment. (…). No, I think just being on my own for a long period of time tends to make one more lonely, and possibly anxious.

(M14 W2)

For many the loss of a sense of belonging was irreplaceable and participants suffered from the loss of loved ones, leaving an unfillable gap. In addition, staying apart from groups and activities they enjoyed increased the gap of belongingness. This gap left many participants missing the resources that family and friends provide in facilitating resilience.

## Discussion

**Fig 3:**
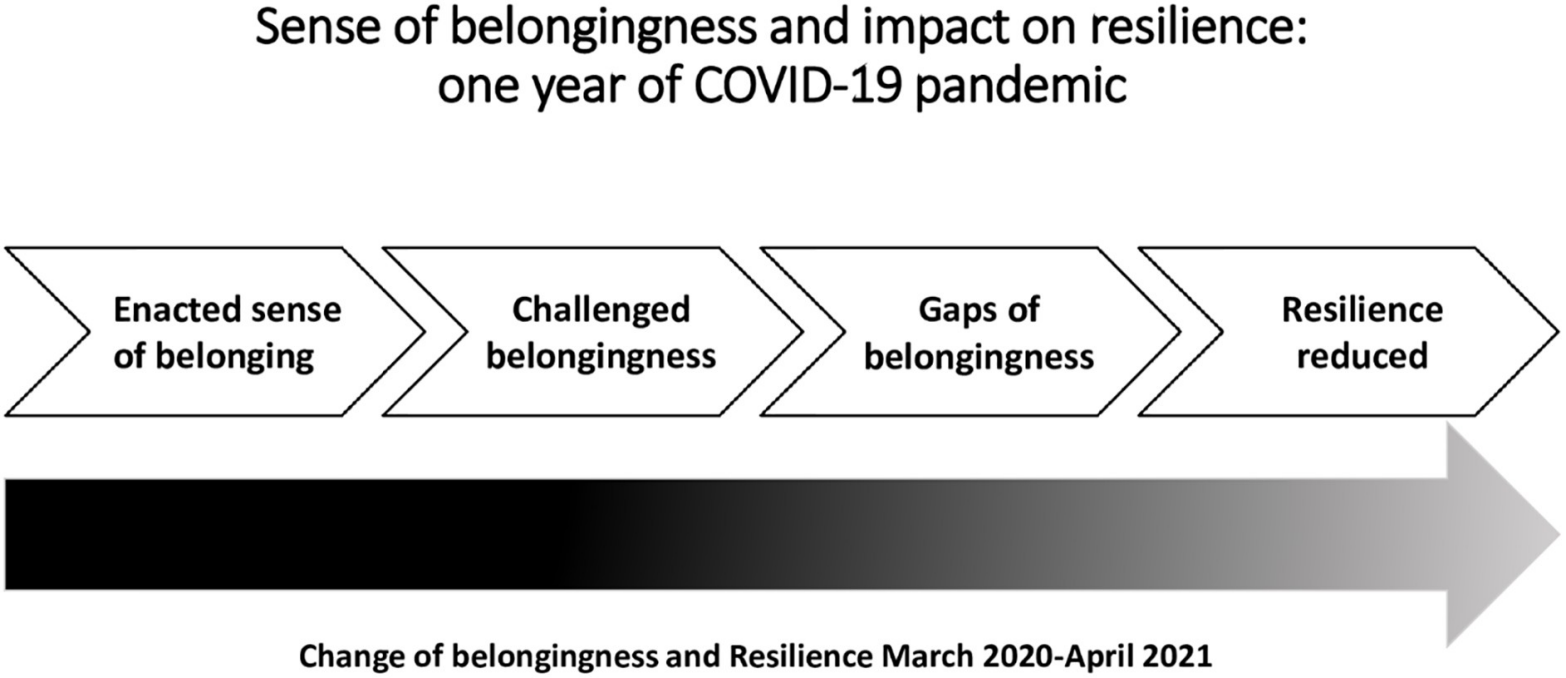
Challenged belongingness and its impact on resilience: First year of COVID-19.

Participants experienced a sense of belonging before the pandemic when they joined family events, met each other, or supported family members via grandparenting. They engaged with friends in cultural or sports activities or found pleasure in going out for meals together. These activities enabled the maintenance of secure bonds and interpersonal relationships [20, 21]. The enacted sense of belonging provided the community and societal resources identified in the ecological model [35] for participants to maintain resilience prior to the pandemic (Fig 2). In March 2020 as the pandemic took hold, the activities and joyful shared experiences were disrupted. The on and off of social distancing measures produced much uncertainty for participants’ lifestyles. However, some were creative and went for a walk whilst social distancing, met outdoors, or drove by to at least see each other. But this did not replace the desired proximity. In consequence, the participants experienced a less joyful time, and the sense of belonging fractured and changed for many from being resilient to not being resilient (Table 1, Fig 3).

Other older adults experienced gaps of belongingness when they withdrew themselves from activities or lost loved ones. The ongoing disruption and gaps of the sense of belonging, persisting uncertainty around the severity of the virus, the unknown individual consequences, and the losses of loved ones impacted people’s well-being. Many participants reported increased anxiety, isolation, frustration, loneliness, feeling of depression, and lower mood. This in turn impacted the older adult’s resilience.

The authors found commonalities amongst participants who changed from being resilient to not being resilient, as well as for participants who were in both interview waves not resilient. First, resources such as community and society (proximity of family, friends, group activities) could not be enacted to foster a sense of belonging and in turn resilience. Second, one year of the COVID-19 pandemic has worn out participants’ resources. Other aspects impacting participants’ resilience like the winter season and the ongoing uncertainty will be published in a later paper.

## Conclusion

Many participants managed to maintain a sense of belonging during the study but were despondent about staying apart from family and friends. They felt that their sense of belonging had been fractured and they were no longer resilient. However, it is important to recognise the heterogeneity of older adults’ sense of belonging and resilience during the time of the COVID-19 pandemic. Our results are important for policy and risk communication both in the light of this pandemic but also in the light of future health threats. Media and communities are required to consider the impact of reporting and communicating risk as well as offering support over a long time of confinement (e.g. providing and distributing contact details for support local, national; planning and establishing a support network). The sense of belongingness needs to be maintained as does social proximity to reduce the risk of poor psychological well-being and to maintain resilience. Future participatory research could investigate how belongingness as a resource for resilience could be maintained in a health crisis.

## Limitations

One limitation of the study was that initial recruitment was via Qualtrics which is an online platform, therefore participants were already online (see McBride *et al*., [52]) for a fuller discussion). Thus, our sample does not include digitally excluded participants. Participants also needed phone or online capabilities to engage in the interview itself, although we were able to provide participants with an MS-Word version of the interview schedule as necessary, and one participant engaged with the study this way. However, one of the advantages of the recruitment was the opportunity to recruit in a more stratified way than would normally be the case with qualitative research. For example, we were able to recruit more men living alone than would often be possible. We were also able to recruit more quickly than would normally be the case, which was especially important in the fast-moving pandemic.

There were potentially additional challenges in conducting interviews remotely. However, in practice, this proved not to be problematic. Rapport was established and distress, on the rare occasions it occurred, was managed effectively with our distress protocol outlined in our ethical approval. This study took place in a western European country and might not reflect the experiences of low-income countries.
